# Laparoscopic appendectomy with single port vs conventional access: systematic review and meta-analysis of randomized clinical trials

**DOI:** 10.1007/s00464-023-10659-w

**Published:** 2024-02-08

**Authors:** Roberto Cirocchi, Maria Chiara Cianci, Lavinia Amato, Luca Properzi, Massimo Buononato, Vanessa Manganelli Di Rienzo, Giovanni Domenico Tebala, Stefano Avenia, Ruggero Iandoli, Alberto Santoro, Nereo Vettoretto, Riccardo Coletta, Antonino Morabito

**Affiliations:** 1 Department of Medicine and Surgery, S. Maria Hospital, University of Perugia, Terni, Italy; 2 Department of Neonatal and Paediatric Surgery, Meyer Children’s Hospital, IRCCS, University of Florence, Florence, Italy; 3 General and Emergency Surgery, S. Maria della Stella Hospital, Orvieto, Italy; 4 Department of Medicine and Surgery, S. Maria Hospital, Perugia, Italy; 5 Digestive and Emergency Surgery Unit, S.Maria Hospital Trust, Terni, Italy; 6 General Surgery P.O. Frangipane Ariano Irpino Asl AV, Ariano Irpino, Italy; 7 Sapienza University of Rome, Rome, Italy; 8 Montichiari Surgery, ASST Spedali Civili, Brescia, Italy

**Keywords:** Acute appendicitis, Laparoscopic appendicectomy, Single-port laparoscopic appendicectomy, Convectional access laparoscopic appendicectomy, Meta-analysis

## Abstract

**Background:**

Conventional three-access laparoscopic appendectomy (CLA) is currently the gold standard treatment, however, Single-Port Laparoscopic Appendectomy (SILA) has been proposed as an alternative. The aim of this systematic review/meta-analysis was to evaluate safety and efficacy of SILA compared with conventional approach.

**Methods:**

Per PRISMA guidelines, we systematically reviewed randomised controlled trials (RCTs) comparing CLA vs SILA for acute appendicitis. The randomised Mantel–Haenszel method was used for the meta-analysis. Statistical data analysis was performed with the Review Manager software and the risk of bias was assessed with the Cochrane "Risk of Bias" assessment tool.

**Results:**

Twenty-one studies (RCTs) were selected (2646 patients). The operative time was significantly longer in the SILA group (MD = 7,32), confirmed in both paediatric (MD = 9,80), (*Q* = 1,47) and adult subgroups (MD = 5,92), (*Q* = 55,85). Overall postoperative morbidity was higher in patients who underwent SILA, but the result was not statistically significant. In SILA group were assessed shorter hospital stays, fewer wound infections and higher conversion rate, but the results were not statistically significant. Meta-analysis was not performed about cosmetics of skin scars and postoperative pain because different scales were used in each study.

**Conclusions:**

This analysis show that SILA, although associated with fewer postoperative wound infection, has a significantly longer operative time. Furthermore, the risk of postoperative general complications is still present. Further studies will be required to analyse outcomes related to postoperative pain and the cosmetics of the surgical scar.

Acute appendicitis is one of the most common abdominal surgical emergencies [1], and conventional three-access laparoscopic appendectomy (CLA) is currently the gold standard treatment [2]. However, an alternative surgical approach, Single-Port Laparoscopic Appendectomy (SILA), has been proposed recently [3, 4]. SILA aims to improve aesthetics, reduce postoperative pain and hospital stay, and thus lead to a faster return to work and improved quality of life. Potential disadvantages of SILA include loss of triangulation, impaired vision, intra/extra abdominal instrument conflicts, and device cost.

Previous literature reviews analysing the results of low-evidence comparative studies (controlled clinical trials—CCTs) have suggested that the two approaches are comparable but have highlighted the need for analyses of randomised controlled trials (RCTs) to suggest which procedure could be the most appropriate [5–7]. Therefore, we performed a systematic literature review and meta-analysis of RCTs to evaluate the safety and efficacy of single-incision laparoscopic appendectomy (SILA) compared with conventional laparoscopic appendectomy.

## Materials and methods

A systematic literature review was conducted until October 2nd 2022, according to the Preferred Reporting Items for Systematic Reviews and Meta-Analyses (PRISMA) guidelines [8]. The research was carried out by analysing the MEDLINE, PubMed, Scopus, and Web of Science databases without language constraints. The registration of protocol was performed on PROSPERO (ID registration CRD42020186856).

Combinations of the following search terms were used: "appendectomy" or "appendectomies, " "single-incision laparoscopic surgery (SILS)", or "single-port laparoscopic surgery", or "single-incision laparoscopic appendectomy", or "conventional 3-port laparoscopic appendectomy" or "conventional laparoscopic surgery" or "multi-incision laparoscopic surgery" or "conventional multiport laparoscopic surgery" or "classic laparoscopic surgery" or "conventional laparoscopic appendectomy" or "single incision" or "single trocar" or "single-port" or "single-port laparoscopic" or "conventional laparoscopic" or "triport laparoscopic" or "one-wound laparoscopic, " and "randomised controlled trial" or "randomised" or "placebo. "

The "Related articles" function of PubMed was used to expand the research and review all eligible studies' reference lists. A manual search was performed through the Google Scholar database to minimise retrieval bias. The search for ongoing clinical studies was performed on ClinicalTrials.gov.

References of all included studies were selected to identify any studies lost during the initial search and were entered into a dataset.

Studies included in this systematic review consider only randomised controlled trials (RCTs) and non-RCTs that have compared conventional laparoscopic appendectomy vs single-port appendectomy for acute appendicitis.

All titles and abstracts were evaluated to identify articles that could be included in the search. Then the full text of these studies was evaluated, and the following information was extracted: year of publication, inclusion criteria, exclusion criteria, and technologies used in the procedure.

The primary outcomes analysed were the following: overall postoperative complications; operative time and incidence of laparotomic conversions.

Secondary outcomes were identified: surgical wound infections; postoperative pain; length of hospital stays, and the cosmetic appearance of skin scars from trocar access holes.

In the analysis of the dichotomous variables, the extracted data were evaluated by Risk Ratio (RR), and in the continuous variables, the data were evaluated by weighted mean differences (WMD) [9].

The randomised Mantel–Haenszel method was used for the meta-analysis. The results obtained were reported in Forest Plot. Higgins index (I^2^), with its 95% confidence intervals and significant levels of Cochrane Q, were considered as indicators of heterogeneity [10].

Statistical data analysis was performed using the Review Manager meta-analysis software (RevMan version 5.4.1) (Copenhagen: The Nordic Cochrane Center, The Cochrane Collaboration, 2018).

Methodological assessment of the risk of bias was performed with the Cochrane "Risk of Bias" assessment tool for randomised control trials (RCTs) [11].

## Systematic review results

**Fig. 1:**
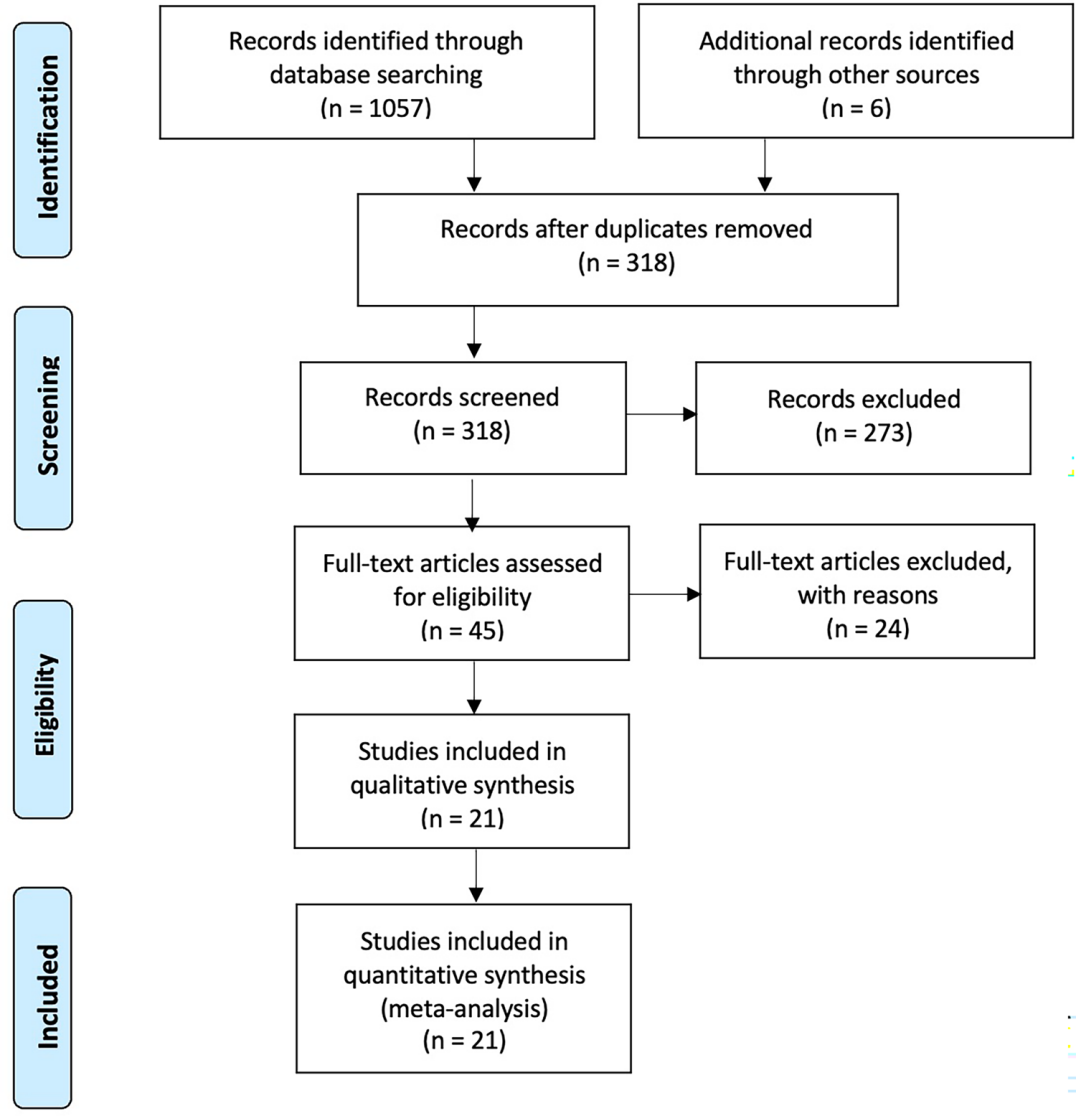
PRISMA flowchart

The PRISMA flow chart for the systematic review is shown in Fig. 1.

The initial literature search identified 1063 potentially relevant articles. After selecting titles and abstracts based on relevance, the remaining 48 articles were further evaluated for eligibility, and 27 others were excluded. The 21 studies included in this meta-analysis are RCTs.

### Characteristics of the studies

The 21 studies enrolled 2646 patients with acute appendicitis who have undergone laparoscopic treatment: 1328 SILA and 1318 CLA. Enrolments took place between 2009 and 2018. Most of the studies were performed in populations from Korea (4 RCTs, comprising a total of 551 patients), the USA (3 RCTs, comprising a total of 485 patients), Spain (3 RCTs, comprising a total of 391 patients) and China (3 RCTs, comprising a total of 339 patients); the remaining studies were conducted in Pakistan (340 patients), Argentina (147 patients), India (100 patients), Scotland (77 patients), Japan (68 patients), Turkey (50 patients), Poland (50 patients) and Egypt (48 patients). Fifteen studies reported surgical experience, but the definitions differed between the included studies.

Thirteen studies reported exclusion criteria such as peri-appendicular abscess and/or mass, generalised peritonitis, appendicular tumour or phlegmon, and perforated appendix; RCTs' criteria were not significantly different.

The patients' ages were heterogeneous, so we performed a subgroup analysis. The clinical characteristics of the patients enrolled in the included studies are summarised in Table 1.

**Table 1:** Clinical characteristics of the patients included in the selected trials

References	No. of patients							
	Single access	Three-port access	Age (years)	Severity of appendicitis (no. of patients)	Local exclusion criteria	Experience of surgical team	Primary outcomes	Follow-up
Shalaby et al. [12]	24	24	12–18	Acute (46), Interval appendectomy (2)	Appendicular abscess or mass, acute appendicitis complicated with generalized peritonitis	NR	NR	NR
Zahara et al. [13]	170	170	15–60	NR	Appendicular tumour and complicated appendectomy	Experienced surgeons	Post-operative pain and surgical site infection	7–15–30 days
Duza et al. [14]	75	72	> 14	Congestive (36), phlegmonous (63), gangrenous (41), appendicular piastron (7)	NR	NR	NR	NR
Golebiewski et al. [20]	25	25	4–17	Early stage, suppurative, gangrenous, perforated, phlegmonous, abscess	Inflammatory tumor or periappendiceal abscess	Experts in both methods	IL-6 and CRP serum concentration	30 days
Moriguchi et al. [15]	34	34	> 8	NR	NR	Experienced and in training surgeons	NR	NR
Mo Kang et al. [21]	90	90	7–71	Uncomplicated, acute	Radiological evidence of perforated appendicitis with local abscess formation or generalized peritonitis	Extensive experience in CLA, minimal experience in SILA	Intraoperative and postoperative morbidity	7 days
Alexander et al. [22]	50	50	> 18	NR	Phlegmon, mass, periappendicular abscess, diffuse peritonitis	NR	NR	NR
Kai Wu et al. [29]	30	30	5–12	Acute (51), perforated (9)	Appendiceal abscess	Experienced surgeons	Surgical outcome of SPLA and CLA using CLA equipment	1, 3, 12 months
Carter et al. [23]	37	38	> 23	Acute (68), Perforated (7)	Phlegmon, mass, periappendicecal abscess, or diffuse peritonitis	Experienced surgeon	Early postoperative pain	2–3 weeks
Mori et al. [24]	60	60	15–65	Without alterations, acute catarrhal, phlegmonous, suppurative, gangrenous	NR	Experts in both methods	NR	6 and 24 months
Frutos et al. [4]	91	93	> 11	Acute (26), phlegmonous (39), purulent (93), gangrenous (26)	Clinical or radiological suspicion of abscess or peritonitis	Experience in advanced laparoscopy and training in SILA	Morbidity	14 days
Kye et al. [26]	51	51	NR	Acute (86), perforated (16)	NR	NR	Pain	20 months
Lee et al. [28]	116	113	> 16	Acute (94), acute suppurative (81), abscess (44), gangrenous (2)	CT or ultrasound positive for abscess	Extensive experience with CLA, > 10 SILA	Morbidity	14 days
Pan et al. [16]	42	42	> 16	Negative inflammation (4), suppurative (39), perforated (12)	Appendicular mass, appendicular abscess	NR	NR	3–12 months
Perez et al. [17]	25	25	3–15	Acute (42), Perforated (8)	NR	Single attending surgeon with assistance of either a pediatric fellow or a senior surgical resident	Operative time	1, 6, 12 months
Scarless et al. [19]	39	38	> 16	NR	NR	Experienced surgeons	Patient-reported outcomes: body image and cosmesis at 6 weeks, clinical outcome: pain at 1–7 days	6 weeks
Sozutek et al. [25]	25	25	> 18	Acute (30), phlegmonous (10), perforated (10)	NR	NR	Pain	30 days
Teoh et al. [27]	98	97	18–75	Normal (7), acute (101) perforated (30), gangrenous (37), abscess (20)	Generalized peritonitis or abscess/ abdominal mass	Performed or supervised by surgeons with experience > 20 SILA and > 100 advanced laparoscopies	Pain	14 days
Villalonga et al. [18]	46	41	> 17	Acute (57), Perforated (28), Chronic (1)	NR	Experienced surgeons	NR	3 months
St. Peter et al. [33]	180	180	< 18	NR	Perforated appendicitis	Experienced surgeon	Postoperative wound infection	6 weeks
Park et al. [31]	20	20	> 25	Acute (39), Perforated (1, intraoperative discovery)	Physical-laboratory-radiological evincence of perforated appendix or periappendiceal abscess	Experienced surgeon	NR	1 week

NR, not recorded; SILA, single-incision laparoscopic appendectomy; CLA, conventional three-port laparoscopic appendectomy

Division of the mesoappendix and section of the base of the appendix was performed with various techniques and tools. The types of single-port and tools used for SILA also varied. The technical characteristics of the surgeries performed in the included studies are summarised in Table 2.

**Table 2:** Technical details of the procedures for single-incision laparoscopic appendectomy (SILA) and conventional three-port laparoscopic appendectomy (CLA)

References	SILA trocar [skin incision length]	Type of instrument for SILA	CLA ports	Stump sealing (CLA and SILA)	Mesoappendix division (CLA and SILA)
Shalaby et al. [12]	Home-made latex surgical glove single port [18 mm]	Dedicated	5 mm trans umbilical 3 mm right upper quadrant 5 mm left lower quadrant	Two home-made endoloops 2/0 vicryl	Monopolar electrocautery
Zahara et al. [13]	Multi- channel port [NR]	Conventional	10 mm intra-umbilical 10 mm port left iliac fossa 5 mm hypogastrium	Endoloop	Diathermy
Duza et al. [14]	One 10 mm port and one 5 mm port (in 31 cases homemade latex gloves,in 6 GelPOINT Mini (Applied Medical), 2 SILS™ Port (Medtronic), in 36 no device) [NR]	Conventional	10 mm umbilical 10 mm soprapubic 5 mm left iliac fossa	Polyglactin suture	Monopolar electrocautery
Golebiewski et al. [20]	SILSPort™ [30 mm]	Dedicated	5 mm umbilical 5 mm soprapubic 5 mm left lower quadrant	Endoloop	Monopolar cautery
Moriguchi et al. [15]	EZ Access port (Hakko) + 2.4 mm percutaneous insertion grasper (MiniLap; Teleflex, Morrisville, NC) placed using puncture technique at the suprapubic region [15 mm]	Dedicated	12 mm umbilical 5 mm left lower abdomen 5 mm suprapubic	Endoloop	Ultrasonically activated device
Mo Kang et al. [21]	Homemade glove port (50 procedures), Octoport® (dalimSurgNET Inc. Seoul, Korea) (40 procedures) [15–30 mm]	Dedicated	10 mm supra/infraumbilical 5 mm left lower quadrant 5 mm suprapubic	NR	NR
Alexander et al. [22]	NR				
Kai Wu et al. [29]	Use of CLA equipment: 3 ports of 5 mm periumbical, later 2 changed to 10 or 12 mm [10 mm, 5 mm]	Conventional	10 or 12 mm umbilical 5 mm left mid abdomen 5 mm left suprapubic	Endoloop	Ultrasonic scalpel
Carter et al. [23]	SILS Port, Covidien [30 mm]	Conventional	12 mm umbilical 5 mm left lower quadrant 5 mm suprapubic midline	Linear stapler or looped suture	Cutting-and-sealing device
Mori et al. [24]	SILS Port, Covidien [25–30 mm]	Dedicated	Hasson Trocar periumbilical 5 mm left iliac fossa 11 mm soprapubic	Endoloop, Endograsp	Endoclip
Frutos et al. [4]	SILS port (Covidien, Mansfield, Massachusetts, USA) [20 mm]	Dedicated	11 mm umbilical 12 mm lower left quadrant 5 mm right upper quadrant	Endoloop	Endostapler
Kye et al. [26]	Home-made glove port [20 mm]	Conventional	10 mm umbilical 5 mm suprapubic 5 mm left lower quadrant	Endoloop	Ultrasonic shears
Lee et al. [28]	Octoport (Dalim,Seoul,Korea) [15 mm]	Conventional	10 mm umbilical 5 mm soprapubic 5 mm left lower quadrant	Endoloop	Ultrasonic shears
Pan et al. [16]	5 mm and 10 mm trocar [15 mm]	Conventional	10 mm umbilical 10 mm right abdomen 5 mm suprapubic	Absorbable ligating clip cartridge	Ultrasound knife
Perez et al. [17]	Triangular orientation at the umbilicus: one 5 mm middle port, two 5 mm lateral ports, later one changed to 12 mm [NR]	Dedicated	NR	NR	Stapler
Scarless et al. [19]	Multi- channel port [NR]	Conventional	10–12 mm intra/supraumbilical 5 or 10 mm left iliac fossa 5 mm hypogastrium	NR	NR
Sozutek et al. [25]	SILS port (Covidien, Mansfield, Massachusetts, USA) [20 mm]	Conventional	10 mm umbilical 5 mm right lower quadrant 5 mm soprapubic	Polypropylene suture	Ultrasonic shears
Teoh et al. [27]	Multiple fascial inserction (two 5 mm ports and one 10 mm port) [13 mm]	Dedicated and conventional	10 mm umbilical 5 mm left lower quadrant 5 mm right lower quadrant	Endoloop	Ultrasonic shears
Villalonga et al. [18]	TriPort (Advanced Surgical Concepts, Wicklow, Ireland) and SILS Port [15–20 mm]	Dedicated	NR	Endoloops	NR
St. Peter et al. [33]	NR [10–20 mm]	Dedicated	12 mm port transumbilical 5 mm left lower quadrant 5 mm suprapubic region	NR	NR
Park et al. [31]	Surgical glove port attached using 3 trocars fixed to the outer ring of a wound retractor [NR]	Dedicated	two 5 mm trocars and a 10 mm trocar	Endoloop	Scalpels or endoclips

NR, not recorded; SILA, single-incision laparoscopic surgery; CLA, conventional three-port laparoscopic appendectomy

The major number of studies do not report the calculation of sample size estimation of patients for randomised controlled trials [12–19]. In few studies the minimal sample size calculation was generically done on the study [20–24]. Differently, other studies [25–28] reported the same outcome on which was performed the sample size calculation, respectively post-operative complications and postoperative pain; only one study [29] reported respectively the operative time as primary outcome. In conclusion the overall field is underpowered to detect a difference in complications between the two techniques—likely due to the fact that complications are rare.

### Study quality assessment

**Fig. 2:**
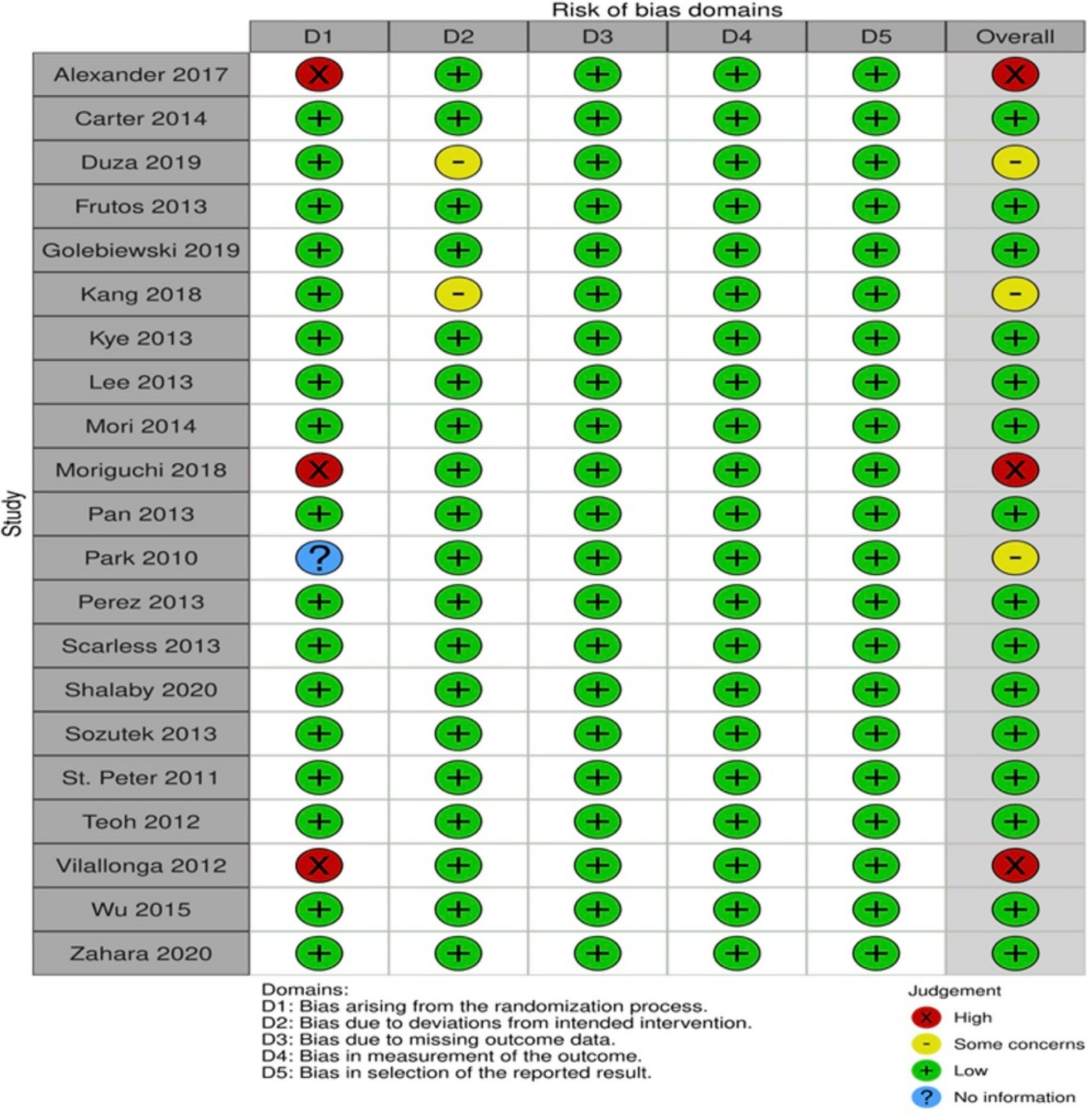
Risk of bias—RCTs

Methodological quality assessment of the RCTs was performed using the RoB 2 (Revised Cochrane risk of bias tool for randomised trials) [30] (Fig. 2). Overall low risk of bias was reported for 71.4% of the studies (15/21), but "blinding" of participants and healthcare personnel was performed in only 2/21 (9.5%) studies [17, 27]. In 14.3% (3/21) of the studies [15, 18, 22], the risk of bias of random sequence generation and allocation was rated as severe, while another 14.3% (3/21) [14, 21, 31] presented some concerns about the risk of bias due to deviation from intended surgeries.

## Meta-analysis results

### Primary outcomes

#### Postoperative complications

**Fig. 3:**
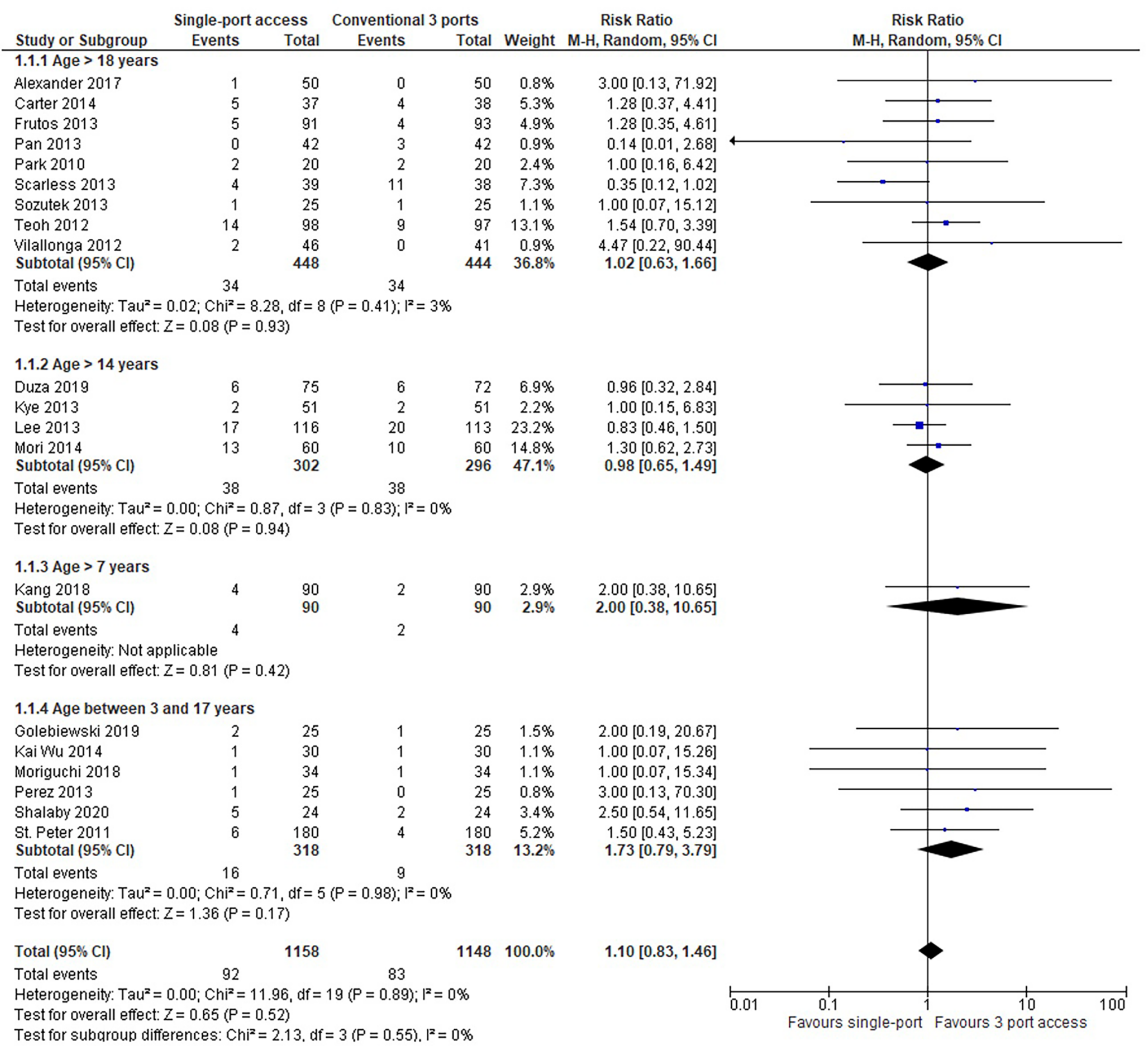
Postoperative complications

In 20 RCTs (2306 patients: 1158 SILA group versus 1148 CLA group), overall postoperative morbidity was higher in patients who underwent SILA (92 patients, 7,94%) compared with CLA (83 patients, 7,22%), but the result was not statistically significant (RR = 1,10, 95% CI 0,83 to 1,46; *P* = 0,52) and heterogeneity was very low (*Q* = 11,96, *P* = 0,89; I2 = 0). Subgroup analysis showed that morbidity was smaller in paediatric patients who underwent SILA (5,03%, 16/318) compared with CLA (2,83%, 9/318), but again the result was not statistically significant (RR = 1,73, 95% CI 0,79 to 3,79; *P* = 0,17) (Fig. 3).

#### Operative time

**Fig. 4:**
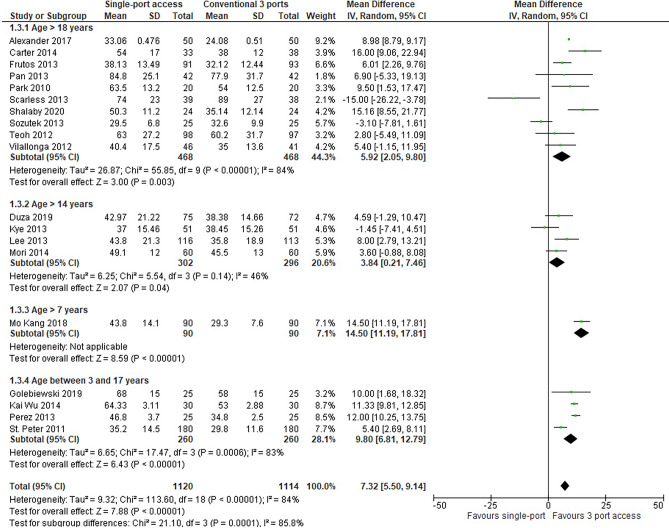
Operative time

In 19 RCTs (2234 patients: 1120 with SILA vs 1114 with CLA group), the operative time was significantly longer in SILA than in CLA (MD = 7,32, 95% CI 5,50 to 9,14; *P* ≤ 0,00001). Heterogeneity was very high (*Q* = 113,60, *P* ≤ 0,00001; I2 = 84%). T he same results reported in the subgroup analysis showed statistically significant favour for the CLA group in paediatric patients (MD = 9,80, IC 95% = 6,81 to 12,79; *P* ≤ 0,00001), (*Q* = 1,47, *P* = 0,00006; I2 = 83%), and in adult patients (MD = 5,92, 95% CI 2,05 to 9,80; *P* = 0,003), (*Q* = 55,85, *P* ≤ 0,00001; I2 = 84%) (Fig. 4).

#### Incidence of laparotomic conversions

**Fig. 5:**
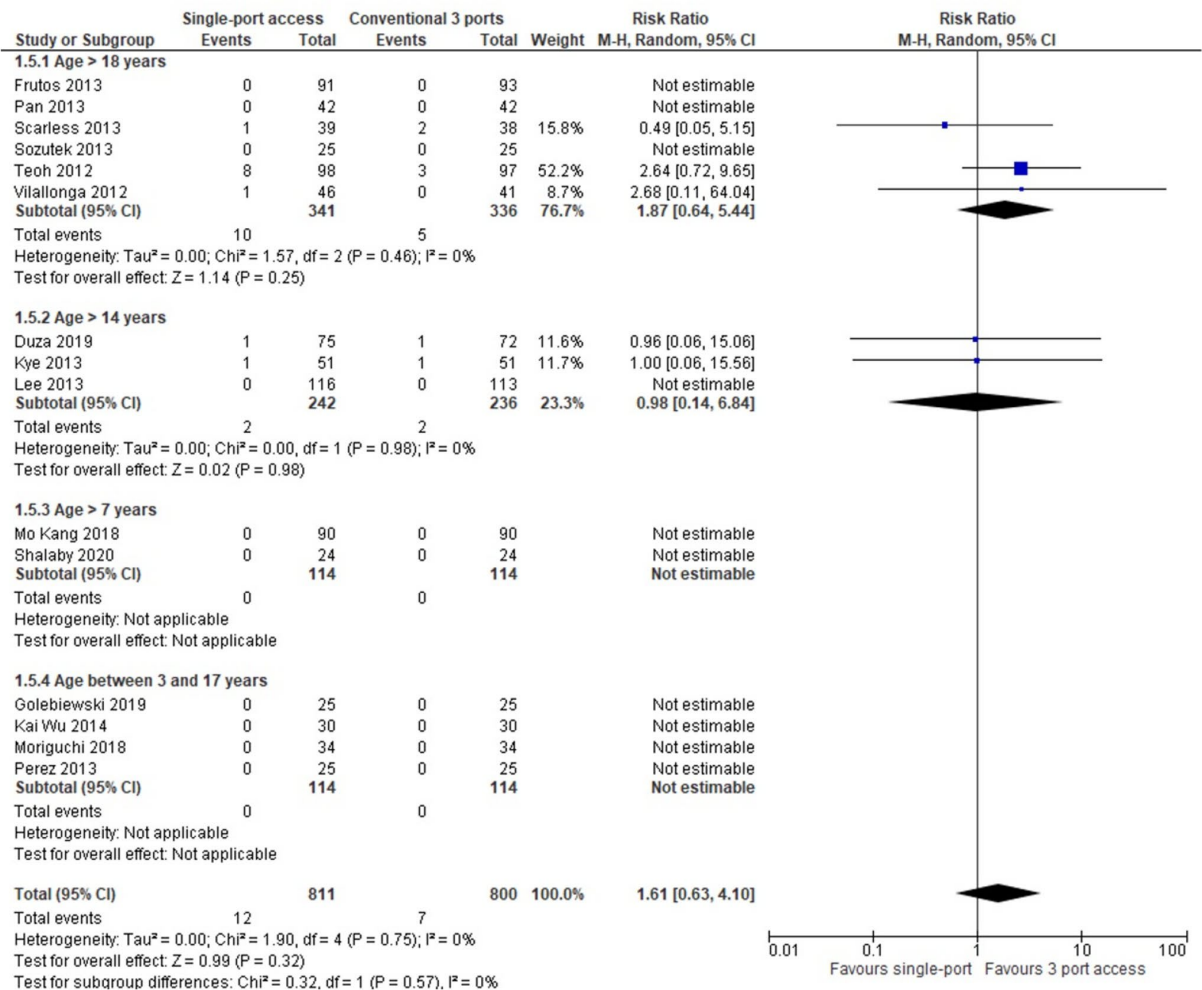
Incidence of laparotomic conversions

In 15 RCTs (1611 patients: 811 in the SILA group vs 800 in the CLA group), a higher rate of laparotomic conversion rate was reported in patients who underwent SILA (12, 1,23%) compared to those who undergone CLA (7 patients, 0,87%) but the result was not statistically significant (RR = 1,53, 95% CI 0,68 to 3,45; *P* = 0,30) and heterogeneity was very low (*Q* = 2,28, *P* = 0,89; I2 = 0%). The result was analysable only in adult patients (Fig. 5).

### Secondary outcomes

#### Surgical wound infections

**Fig. 6:**
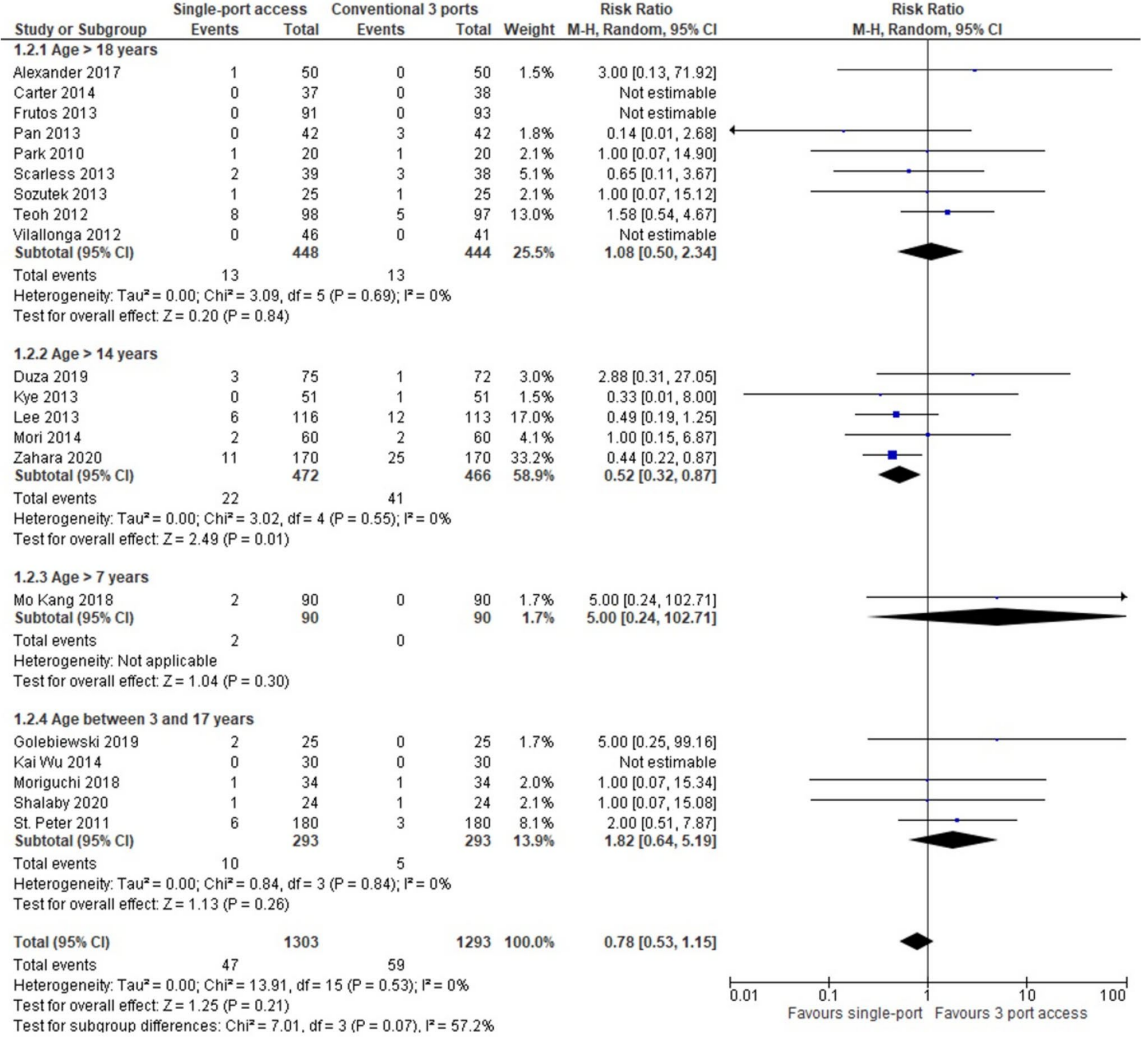
Surgical wound infections

In 20 RCTs (2596 patients: 1303 underwent SILA vs 1293 underwent CLA), postoperative wound infections were lower in patients who underwent SILA (47 patients, 3,6%) than in those who underwent CLA (59 patients, 4,56%), but the result was not statistically significant (RR = 0,78, 95% CI 0,53 to 1,15; *P* = 0,21). Heterogeneity was very low (*Q* = 13,91, *P* = 0,53; I2 = 0) (Fig. 6).

#### Length of hospital stay

**Fig. 7:**
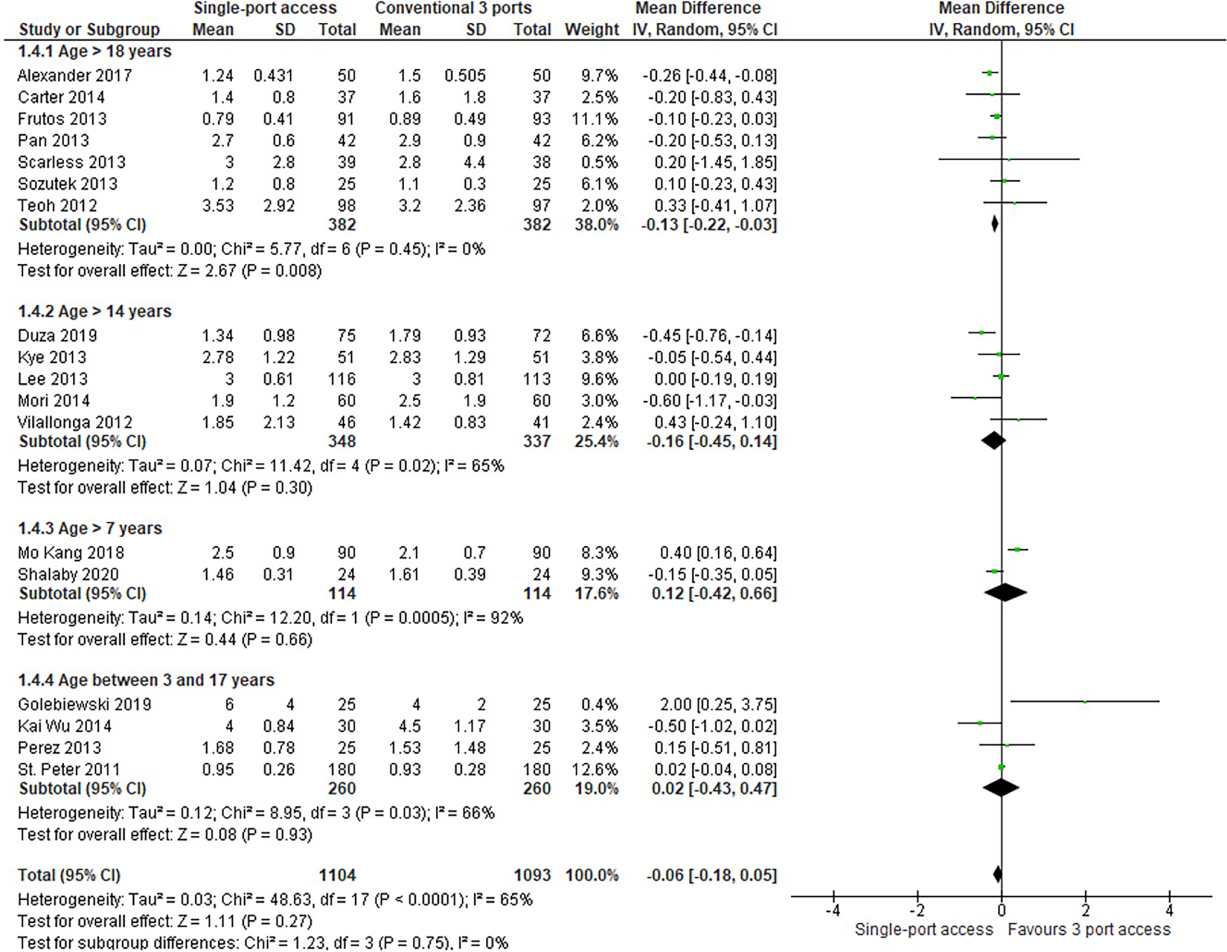
Length of hospital stay

Length of hospital stay was reported in 18 RCTs (2197 patients: 1104 underwent SILA vs 1093 underwent CLA). The analysis showed a shorter hospital stay in patients who underwent SILA, but the result was not statistically significant (MD = − 0,006, 95% CI − 0,18 to 0,05; *P* = 0,27); heterogeneity was very high (*Q* = 48,63, *P* = 0,0001; I2 = 65%) (Fig. 7).

#### Postoperative pain

Sixteen studies reported results on postoperative pain, but we did not summarise the results because different scales and assessment times were used (Table 3) [4, 12–29, 31, 33].

**Table 3:** Comparison between different scales of postoperative pain

Study	Scale	Evaluation after surgery	Laparoscopic appendectomy (mean $\pm$ SD)		
			CLA	SILA	*P*
Shalaby et al. [12]	NR		NR	NR	NR
Zahara et al. [13]	VAS (NR)	At 24 h	4.08 ± 1.382	3.09 ± 1.477	0.0001
Duza et al. [14]	VAS (value NR)	At 12 h At discharge	5.31 $\pm 2.93$ 3.87 $\pm 2.02$	3.93 $\pm 1.92$ 2.33 $\pm 1.32$	0.000 0.0001
Golebiewski et al. [20]	VAS (graded from 0 to 10)	NR	3 $\pm 1.5$	5 $\pm 2$	0.001754
Moriguchi et al. [15]	NR		NR	NR	NR
Mo Kang et al. [21]	VAS (graded from 0 to 7)	At 12 h At 24 h At 36 h	NR	NR	NR
Alexander et al. [22]	VAS (NR)	At 6 h At 12 h At 24 h	1.16 ± 0.374 1.88 ± 0.332 1.00 ± 0.000	1.00 ± 0.000 1.36 ± 0.490 1.00 ± 0.000	
Kai Wu et al. [29]	NR	NR	NR	NR	NR
Carter et al. [23]	NR	During first 12 h	3.5 ± 1.5	4.4 ± 1.6	0.01
Mori et al. [24]	VAS (graded from 0 to 10)	NR	3.3 $\pm 0.5$	3.9 $\pm 1.3$	0.0004
Frutos et al. [4]	VAS (graded from 0 to 10)	At 9 h and 12 h	3.78 $\pm$ 1.76	2.76 $\pm 1.64$	< 0.001
Kye et al. [26]	VAS (value NR)	At 24 h At 48 h	3.22 $\pm 1.22$ 2.20 $\pm 1.03$	3.22 $\pm 1.22$ 2.04 $\pm 1.12$	0.012 0.460
Lee et al. [28]	VAS (value NR)	At 12 h At 24 h At 36 h At 14 days	NR		0.651 0.555 0.570 0.631
Pan et al. [16]	NR	NR	NR	NR	NR
Perez et al. [17]	NR	NR	NR	NR	NR
Scarless et al. [19]	NR	At 1–7 days At 6 weeks	22.4 $\pm 10.8$ 0.6 $\pm 1$	19.4 $\pm 11.9$ 0.4 $\pm 0.7$	0.36 0.47
Sozutek et al. [25]	VAS (graded from 0 to 10)	At 3 h At 6 h At 12 h At 24 h	5.1 $\pm 1.2$ 3.4 $\pm 1.0$ 2.1 $\pm 0.81$ 2.0 $\pm 1.0$	4.4 $\pm 1.1$ 2.89 $\pm 0.86$ 2.1 $\pm 0.97$ 2.0 $\pm 0.95$	0.001 0.001 0.001 0.078
Teoh et al. [27]	VAS (graded from 0 to 100)	At 24 h	NR		0.253
Villalonga et al. [18]	VAS (NR)	At 12 h	2.9 ± 0.78	2.8 ± 0.9	0.774
St. Peter et al. [33]	NR	NR	NR	NR	NR
Park et al. [31]	VAS (graded from 0 to 10)	At 1–2 days	NR	NR	NR

VAS, visual analogue scale; NR, not reported; CLA, conventional three-port laparoscopc appendectomy; SILA, single incision laparoscopic appendectomy

#### The aesthetic appearance of skin scars of trocar access holes

Only nine studies reported results on the cosmetic appearance of skin scars of trocar access holes, but meta-analysis was not performed because different scales were used in each study (Table 4) [14, 16, 18, 19, 21, 23, 25, 27, 31].

**Table 4:** Comparison between different scales of postoperative cosmesis

Study	Scale of cosmesis score	Evaluation	Laparoscopic appendectomy (mean $\pm {\text{SD}})$		
			CLA	SILA	*P*
Shalaby et al. [12]	NR	NR	NR	NR	NR
Zahara et al. [13]	NR	NR	NR	NR	NR
Duza et. al [14]	VSS	NR	7.61 $\pm 1.34$	9.54 $\pm 0.94$	0.07
Golebiewski et al. [20]	NR	NR	NR	NR	NR
Moriguchi et al. [15]	NR	NR	NR	NR	NR
Mo Kang et al. [21]	NR	3 months	NR	NR	NR
Alexander et al. [22]	NR	NR	NR	NR	NR
Kai Wu et al. [29]	NR	NR	NR	NR	NR
Carter et al. [23]	VAS	6 months	16.4 $\pm$ 3.0	18.4 $\pm$ 2.7	0.01
Mori et al. [24]	NR	NR	NR	NR	NR
Frutos et al. [4]	NR	NR	NR	NR	NR
Kye et al. [26]	NR	NR	NR	NR	NR
Lee et al. [28]	NR	NR	NR	NR	NR
Pan et al. [16]	NR	3–12 months	3.9 $\pm 0.9$	4.5 $\pm 0.7$	0.001
Perez et al. [17]	NR	NR	NR	NR	NR
Scarless et al. [19]	NR	At 6 weeks	NR	NR	NR
Sozutek et al. [25]	NR	At first month	6.7 $\pm 0.8$	7.2 $\pm 0.8$	0.247
Teoh et al. [27]	Rating from 0 to 100	At 2 weeks	73.43 $\pm 24.09$	82.50 $\pm 20.17$	0.002
Villalonga et al. [18]	NR	NR	7.4 $\pm$ 1.3	8.6 $\pm 0.9$	0.001
St. Peter et al. [33]	NR	NR	NR	NR	NR
Park et al. [31]	NR	At 7 day	NR	NR	NR

NR, not recorded; CLA, conventional three-port laparoscopic appendectomy; SILA, single incision laparoscopic appendectomy

## Discussion

This systematic review of the literature pooled the results of 21 RCTs (2646 patients enrolled). It showed that SILA is comparable to CLA in treating acute appendicitis and may have some benefits.

The most important limitation of SILA is reported in the characteristics of thirteen included, studies that reported exclusion criteria as appendicular phlegmon, perforated appendicitis and so on. Very probably this limitation could influence outcomes especially in technically difficult cases. For this reason, SILA needed an extreme accurate selection of patients: uncomplicated appendicitis associated at mild inflammatory status. In fact, the feasibility of SILA can be very low in complicated appendicitis and it is associated with higher rate risk of conversion to laparotomy and at the impossibility to place an abdominal drainage tube [32].

The only significant finding in this analysis are relates to the longer operative time, which increases significantly for SILA, as observed in most single-incision surgeries. In fact, because of intra/extra abdominal instrument conflicts, SILA is relatively difficult to perform, resulting in a significantly longer operative time than CLA. In some studies, a single surgeon performed the procedures [23, 31, 33], while other methods involved multiple surgeons. Due to differences in surgical experience, this may have influenced the statistical results. Furthermore, this longer operative time can be the consequence of limited manpower and extra time needed for the preparation and manipulation of the camera holder [34]. This longer operative time is associated at higher cost [35] and major postoperative pain for muscular stretching at the single umbilical wound [36].

Data regarding overall morbidity showed fewer cases in the CLA (7,22%) group than in the SILA group (7,94%), an advantage especially evident in paediatric patients. However, they showed no statistically significant difference between SILA and CLA (RR = 1,10, 95% CI 0,83 to 1,46; *P* = 0,52). In contrast, data analysis regarding abdominal wall morbidity, although equally nonsignificant, suggested a lower incidence of postoperative surgical wound infections in patients who underwent SILA. Therefore, SILA can be considered a safe and effective technique.

Hospital stay was lower in patients who underwent SILA. At the same time, the incidence of laparotomy conversions was lower in CLA than in SILA, but the results were not statistically significant in both cases. This heterogeneity of hospital stay is associated with the different timing of oral intake after appendectomy. Previously, the oral intake starts at return of bowel function evaluated during abdominal examination (bowel sound and passage of flatus); differently in other surgical unit with adopted the ERAS program a soft diet is performed as soon as possible (5–7 h post-surgery) independently from the bowel movement [37].

Data on pain were not summarised because of the heterogeneity of scales and assessment times. Although influenced by the above considerations, the RCTs did not show a significantly better outcome regarding postoperative pain or a reduction in the need for analgesics. The same applies to cosmetics data, which are not homogeneous and have yet to be grouped in a forest plot. Cosmetics were assessed in multiple of our studies through fill-in questionnaires based on subjective judgment and not through international rating scales, making cosmetic appearance an impossible outcome to compare.

The financial cost was not compared in the studies included in our meta-analysis. However, it has been pointed out that SILA can be safely performed with the same tools and costs as CLA [29]. High-energy dissection instruments, dedicated angled instruments and commercial single-access port devices were widely used in this study, which, having a high cost, could influence the choice between the two operations. The use of conventional instruments, bipolar coagulation for mesoappendix and limited application of endo stapler for stump transection would significantly lower the cost of the operation. In addition, the solution adopted by Kye et al. [26], Duza et al. [14], Shalaby et al. [12], Mo Kang et al. [21], and Park et al. [31] considers the use of a "homemade" port with a latex surgical glove, which would not affect the budget, unlike commercial single-access ports.

A limitation of the analysis is the need for more data examining the learning curve for SILA. In the studies included in our review, the specification of prior training in SILA was quantified only by Teoh et al. [27] and Lee et al. [28], who considered at least 20 and 10 SILA procedures, respectively, to be necessary to ensure competence in SILA. Frutos et al. [4] and Moriguchi et al. [15] described "previous training in SILA" without further specification, as did Mo Kang et al. [21], which reported only minimal experience in SILA. Golebiewsky et al. [20] and Mori et al. [24] described their experience with both surgical techniques. In an analysis of learning curve, Kim suggested that surgeons can have an adequate surgical skills for SILA after performed 30 appendectomy, furthermore other 90 appendectomy are needed to gain an experienced surgical skills [38].

The present meta-analysis is also limited because the results collected were mainly short-term indicators and needed long-term follow-up results. One of these long-term outcomes is port-site incisional hernia. In fact the literature reported a high rate of incisional hernia rate. In a recent systematic review of literature and meta-analysis performed on single-incision laparoscopic, surgery is associated with a threefold increase in the odds of incisional hernia than conventional laparoscopic surgery (odds ratio 2,83, 95% CI 1,34–5,98, *P* = 0,006, I2 = 0%) [39], similar results were reported from previous review [40, 41]. For this reason, recommendation to prevent incisional hernias is commonly performed [42].

Moreover, the results included subjective indices such as pain and aesthetic scores, which patients and evaluators easily influence.

## Conclusions

In conclusion, this systematic review and meta-analysis show that Single-Port laparoscopic appendectomy do not report any advantage for the SILA, but there is only significantly longer operative time than CLA. Furthermore, it is not free from the risk of postoperative general complications, prolonged hospital stays, and the need for conversion. Probably, the inferiority of SILA than CLA and the complex learning curve are the reasons for which the SILA is uncommon.

Further studies will be required to analyse outcomes related to postoperative pain and the cosmetics of the surgical scar and make cost–benefit assessments.

## Data Availability

The data used to support the finding of this study are included within the article.
