# Correction: Laparoscopic appendectomy with single port vs conventional access: systematic review and meta-analysis of randomized clinical trials

**DOI:** 10.1007/s00464-024-10750-w

**Published:** 2024-02-20

**Authors:** Roberto Cirocchi, Maria Chiara Cianci, Lavinia Amato, Luca Properzi, Massimo Buononato, Vanessa Manganelli Di Rienzo, Giovanni Domenico Tebala, Stefano Avenia, Ruggero Iandoli, Alberto Santoro, Nereo Vettoretto, Riccardo Coletta, Antonino Morabito

**Affiliations:** 1 Department of Medicine and Surgery, S. Maria Hospital, University of Perugia, Terni, Italy; 2 Department of Neonatal and Paediatric Surgery, Meyer Children’s Hospital, IRCCS, University of Florence, Florence, Italy; 3 General and Emergency Surgery, S. Maria della Stella Hospital, Orvieto, Italy; 4 Department of Medicine and Surgery, S. Maria Hospital, Perugia, Italy; 5 Digestive and Emergency Surgery Unit, S.Maria Hospital Trust, Terni, Italy; 6 General Surgery P.O. Frangipane Ariano Irpino Asl AV, Ariano Irpino, Italy; 7 Sapienza University of Rome, Rome, Italy; 8 Montichiari Surgery, ASST Spedali Civili, Brescia, Italy

**Correction to: Surgical Endoscopy** 10.1007/s00464-023-10659-w

The original online version of this article was revised to correct the presentation of affiliation 2. The correct affiliation is:

Department of Neonatal and Paediatric Surgery, Meyer Children’s Hospital, IRCCS, University of Florence, Florence, Italy.

The original article has been corrected.

